# Embolic Stroke Unveiling Lung Cancer: A Case of Cancer-Associated Non-bacterial Thrombotic Endocarditis

**DOI:** 10.7759/cureus.78339

**Published:** 2025-02-01

**Authors:** Daniela Barbosa Mateus, Tiago Jesus

**Affiliations:** 1 Internal Medicine, Hospital Vila Franca de Xira, Vila Franca de Xira, PRT; 2 Neurology, Hospital Vila Franca de Xira, Vila Franca de Xira, PRT

**Keywords:** cardioembolism, embolic stroke, ischemic stroke, lung cancer, non-bacterial thrombotic endocarditis

## Abstract

We discuss the case of a 71-year-old male with various comorbidities who presented to the Emergency Department for prostration and gait abnormalities. Neurological assessment and imaging studies revealed a multi-territorial ischemic stroke. Further investigation identified a cardiac mass, raising suspicion of non-bacterial thrombotic endocarditis (NBTE), after ruling out infectious causes. Further investigation, a thoracoabdominopelvic computed tomography (CT) scan revealed a pulmonary mass, confirmed as non-small cell lung carcinoma. The final diagnosis was cardioembolic stroke secondary to NBTE, associated with a hypercoagulable state induced by malignancy. The patient was started on therapeutic anticoagulation and referred for multidisciplinary management.

This case underscores the significance of NBTE in stroke cases, highlighting the importance of early consideration of malignancy. Early identification is crucial for prompt initiation of appropriate treatment.

## Introduction

Cardioembolism accounts for approximately 20% to 40% of all ischemic strokes annually. High-risk sources include atrial fibrillation, valvular disease, severe left ventricular dysfunction, endocarditis, cardiac tumors, paradoxical embolism, and intracardiac thrombi [1].

Non-bacterial thrombotic endocarditis (NBTE) is a rare entity characterized by sterile vegetations on cardiac valves, primarily composed of fibrin and platelets. This condition is strongly associated with hypercoagulable states, often secondary to malignancies. Ischemic stroke may represent the initial presentation of an underlying neoplastic process driven by the pro-thrombotic environment these conditions create [2]. This article reports the case of a patient with a multi-territorial ischemic stroke caused by NBTE, which was ultimately traced to an underlying lung malignancy.

## Case presentation

A 71-year-old male with a medical history of type 2 diabetes mellitus complicated by nephropathy and retinopathy, hypertension, benign prostatic hyperplasia, hyperuricemia with poor therapeutic adherence, significant alcohol consumption (10 grams/day), a 40-pack-year smoking history, and a past diagnosis of Q fever treated with doxycycline three years ago, presented to the Emergency Department with a one-week history of prostration and gait disturbances.

On examination, the patient presented with right homonymous hemianopia, mild left central facial palsy, very mild left hemiparesis, a left-sided extensor plantar reflex, and gait ataxia, with a National Institutes of Health Stroke Scale (NIHSS) score of 4. Auscultation revealed a grade II systolic murmur audible at all cardiac foci. Hemodynamically stable, afebrile, eupneic with adequate peripheral oxygen saturation without oxygen therapy, heart rate of 70 bpm, and blood pressure of 152/96 mmHg.

Laboratory findings at admission (Table 1) included normocytic normochromic anemia (11.3 g/dL), leukocytosis (11,600/μL) with neutrophilia (9,310/μL), elevated C-reactive protein (5.74 mg/dL), urea (87 mg/dL), creatinine (2.17 mg/dL), markedly elevated D-dimers (15,432 ng/mL) and high-sensitivity troponin I (1,323 pg/mL).

**Table 1:** Laboratory findings on the day of admission to the emergency room.

Test	Value	Reference range
Hemoglobin	11.3 g/dL	13.0-16.9 g/dL
White blood cells	11,600/μL	4,000-11,000/µL
C-reactive protein	5.74 mg/dL	< 0.5 mg/dL
Urea	87 mg/dL	< 50 mg/dL
Creatinine	2.17 mg/dL	0.7-1.3 mg/dL
D-dimers	15,432 ng/mL	< 500 ng/mL
High-sensitivity troponin I	1,323 pg/mL	< 72 pg/mL

**Figure 1:**
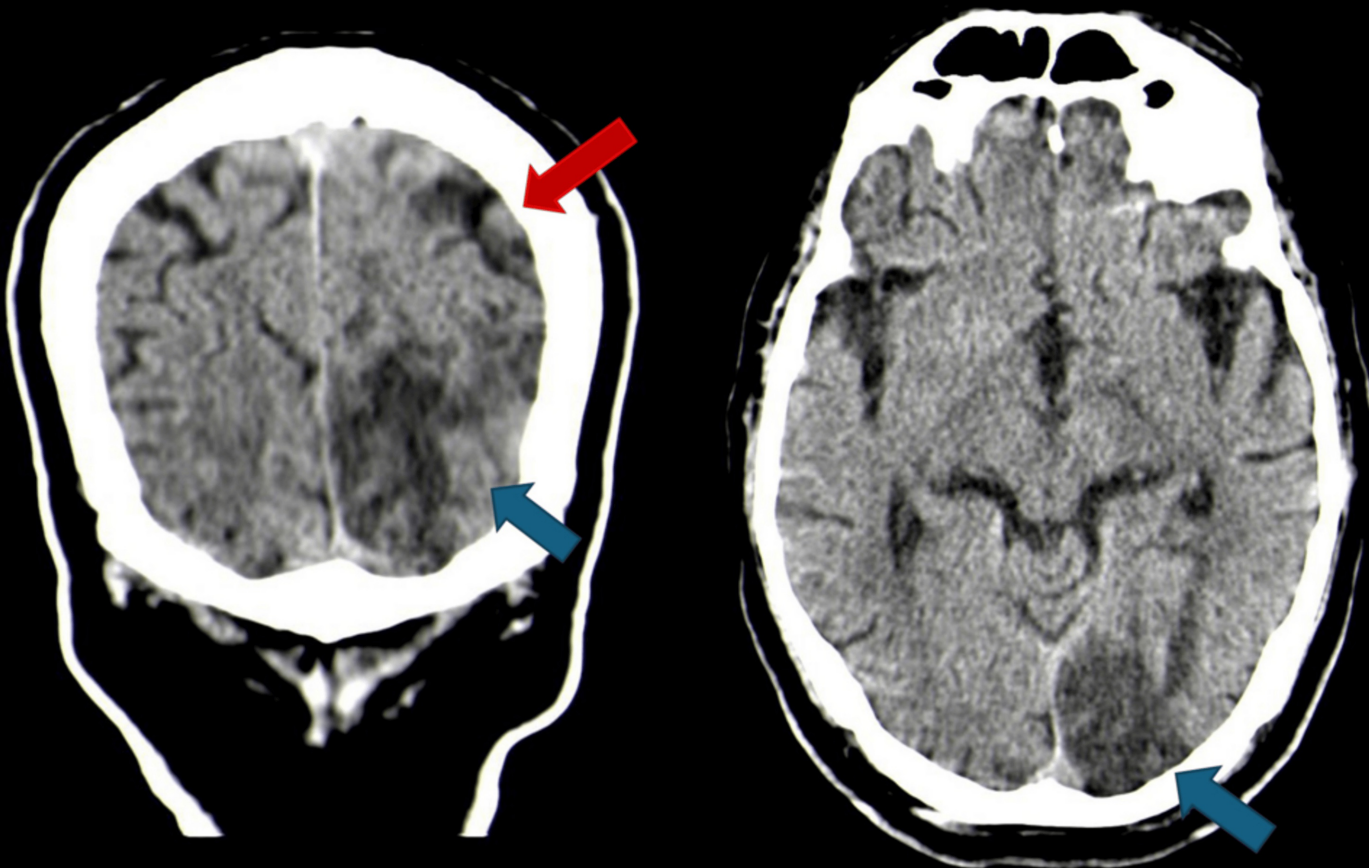
Cranial CT (coronal plane on the left and axial plane on the right): the blue arrow indicates an occipital infarction, while the red arrow highlights a small cortical parietal infarction in a different arterial territory.

**Figure 2:**
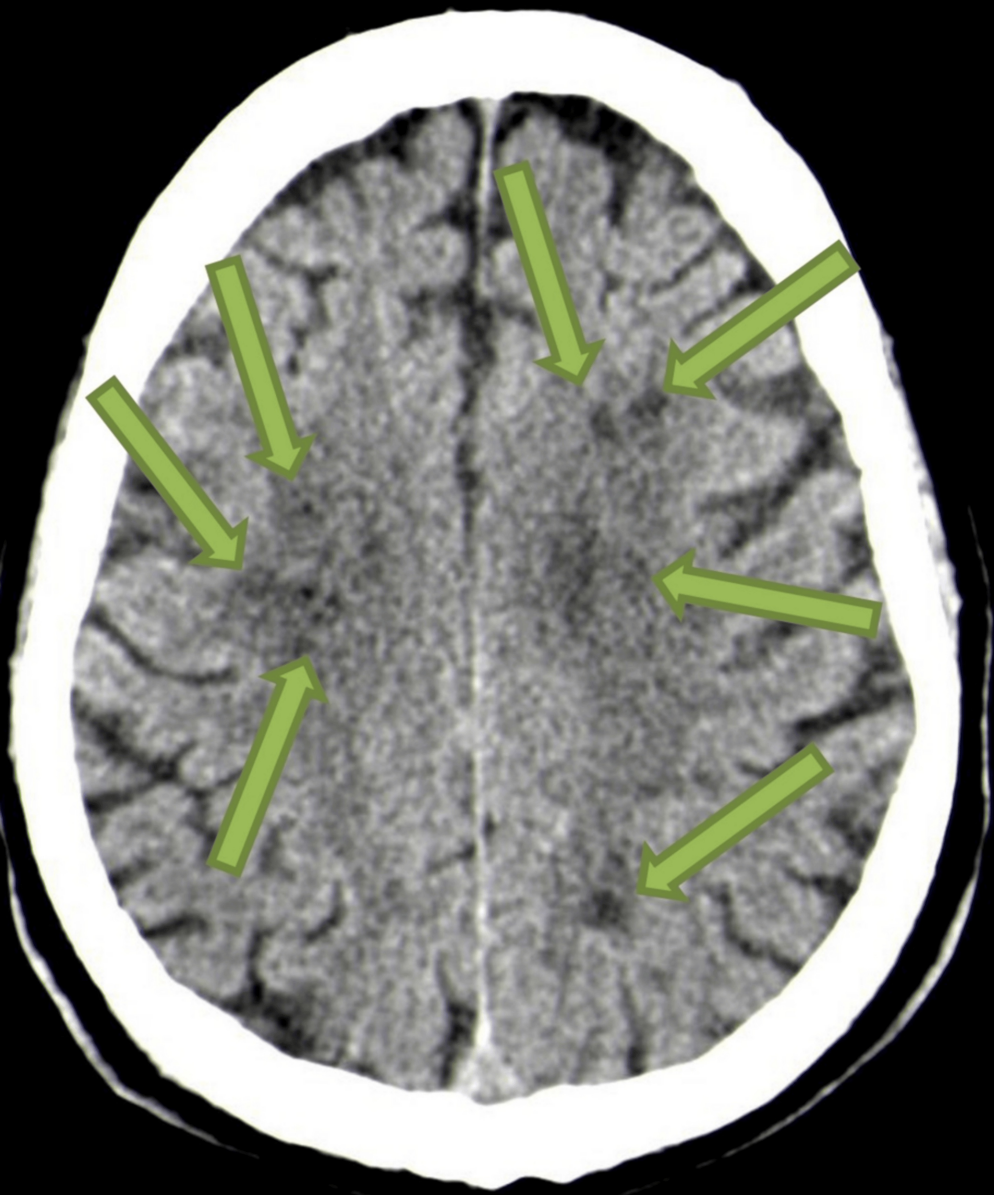
Cranial CT (axial plane): the green arrow indicates small focal lesions in the centrum semiovale.

Cranial computed tomography (CT) revealed cortical-subcortical hypodensities in the left temporo-occipital and parasagittal parietal regions, consistent with subacute infarction in the posterior cerebral artery territory with additional small focal lesions in the centrum semiovale (Figures 1, 2).

**Figure 3:**
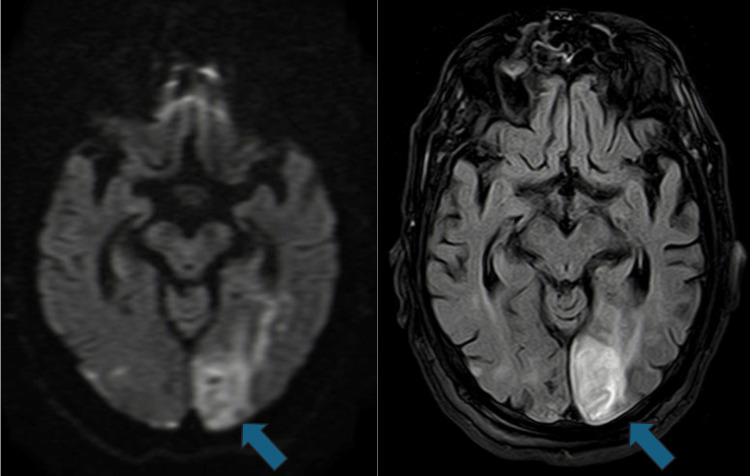
Brain MRI (DWI (left) and FLAIR (right) axial views): left occipital infarction and small contralateral occipital cortical lesions. DWI: Diffusion-Weighted Imaging, FLAIR: Fluid-Attenuated Inversion Recovery

**Figure 4:**
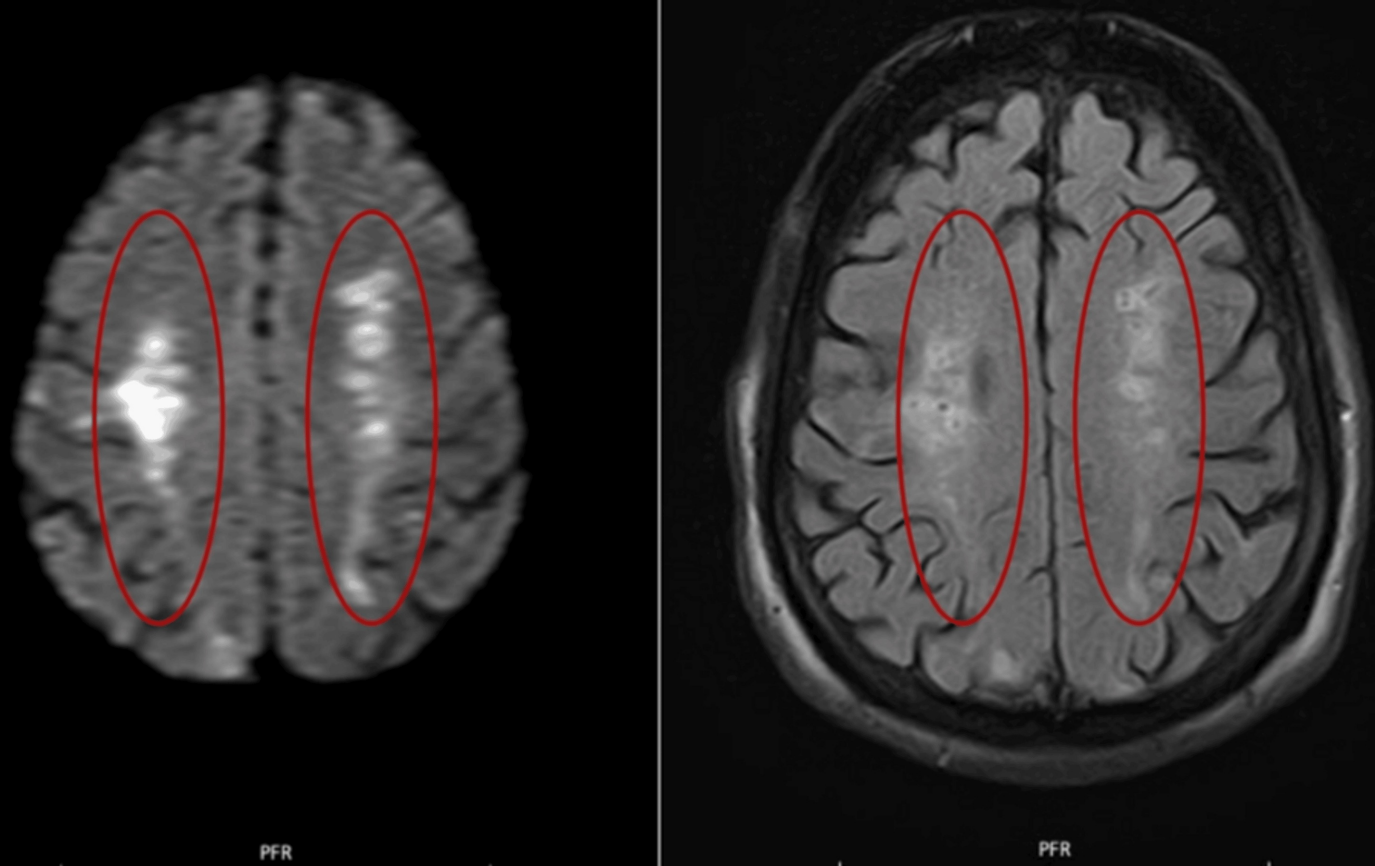
Axial DWI (left) and FLAIR (right) images – multiple recent ischemic lesions in the centrum semiovale, forming a bilateral "line" pattern, suggestive of watershed stroke. DWI: Diffusion-Weighted Imaging, FLAIR: Fluid-Attenuated Inversion Recovery

Subsequent magnetic resonance imaging (MRI) confirmed multiple areas of restricted diffusion and fluid-attenuated inversion recovery (FLAIR) hyperintensities in both anterior and posterior circulation territories. These findings were consistent with microembolic, multi-territorial ischemic lesions (Figures 3, 4).

Electrocardiography revealed sinus rhythm with no evidence of acute ischemic changes. A 24-hour Holter monitor confirmed sinus rhythm without any significant abnormalities. Telemetry during hospitalization consistently showed sinus rhythm.

**Figure 5:**
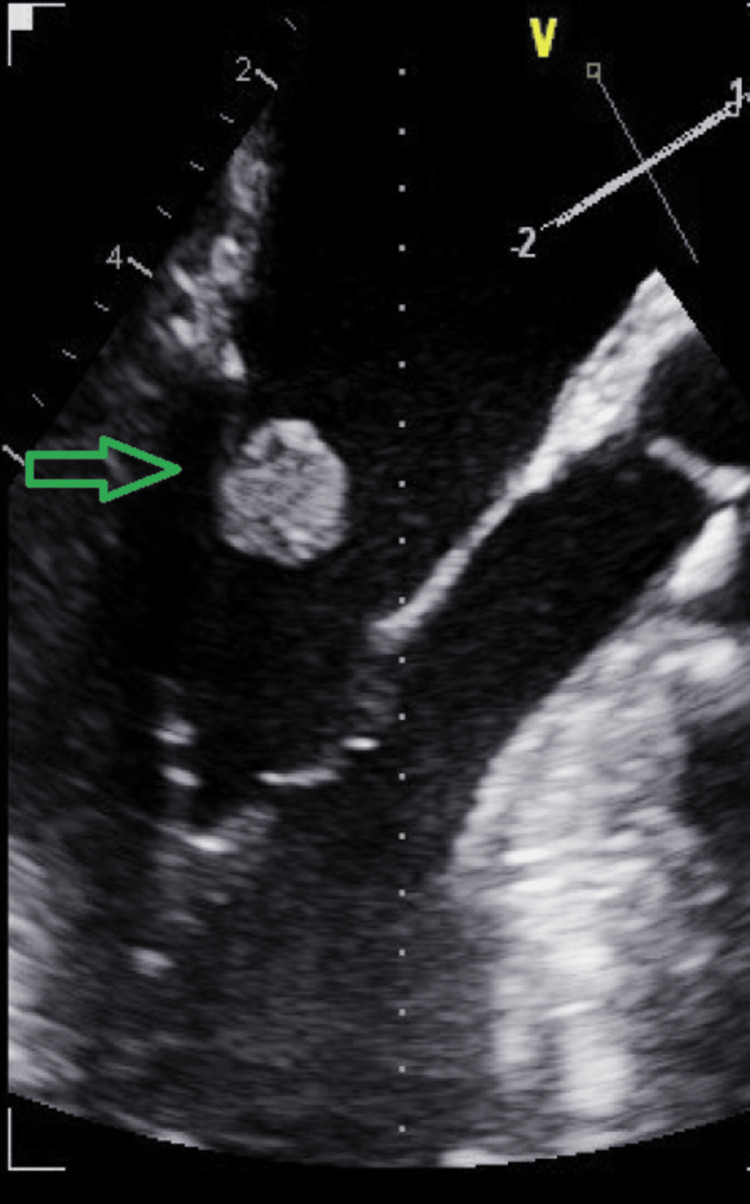
Transesophageal echocardiography showing a heterogeneous mass on the mitral valve.

Transthoracic echocardiography identified a heterogeneous mass on the atrial side of the posterior mitral leaflet (13 × 12 mm), raising suspicion of vegetation or a tumoral mass. Subsequent transesophageal echocardiography revealed a 10.6 × 10.3 mm heterogeneous, rounded lesion adherent to the atrial surface of the posterior mitral leaflet, consistent with vegetation. Additional findings included mild mitral, tricuspid, and pulmonary regurgitation, a non-dilated left atrium and appendage without thrombi, and a non-dilated left ventricle with preserved systolic function (Figure 5).

Additional vascular studies yielded unremarkable results, including carotid, vertebral, and transcranial Doppler. Despite the early identification of the embolic source, its etiology remained unclear. To rule out infectious causes, three sets of blood cultures were obtained on different days (day 0, day 5, and day 17), all of which were negative. C-reactive protein levels remained stable, peaking at admission (5.74 mg/dL), without leukocytosis, and the patient remained afebrile throughout hospitalization. Considering a prior history of Q fever, serological testing was repeated, revealing values consistent with the convalescent phase and excluding chronic infection. Additional diagnostic tests included a negative interferon-gamma release assay (IGRA), non-reactive Venereal Disease Research Laboratory (VDRL), and non-reactive HIV 1/2 (Table 2).

**Table 2:** Significant laboratory findings throughout the hospitalization. VDRL: Venereal Disease Research Laboratory, IGRA: Interferon-Gamma Release Assay, PSA: Prostate-Specific Antigen

Test	Value	Reference range
VDRL	Non-reactive	
HIV 1/2	Non-reactive	
High-sensitivity troponin I 24h later admission	1401 pg/mL	< 72 pg/mL
Anti-Coxiella burnetti phase I IgG antibodies	Negative (< 1/64)	< 1/64
Anti-Coxiella burnetti phase I IgM antibodies	Negative (< 1/24)	< 1/24
Anti-Coxiella burnetti phase II IgG antibodies	Positive (1/64) 3 years before: 1/1024	< 1/64
Anti-Coxiella burnetti phase II IgM antibodies	Positive (1/24) 3 years before: 1/384	< 1/24
IGRA	0.03 UI/mL	< 0.35 UI/mL
Alpha-fetoprotein	<1.30 ng/mL	< 8.1 ng/mL
PSA level	1.85 ng/mL	< 6.5 ng/mL
C-reactive protein variation	3.16 – 5.74 mg/dL	< 1 mg/dL

According to the modified Duke criteria, the case met one major criterion (imaging evidence of vegetation on the mitral valve) and one minor criterion (ischemic stroke compatible with bilateral embolic phenomena). This was insufficient to establish a diagnosis of infectious endocarditis and could also be consistent with non-infectious endocarditis [3].

**Figure 6:**
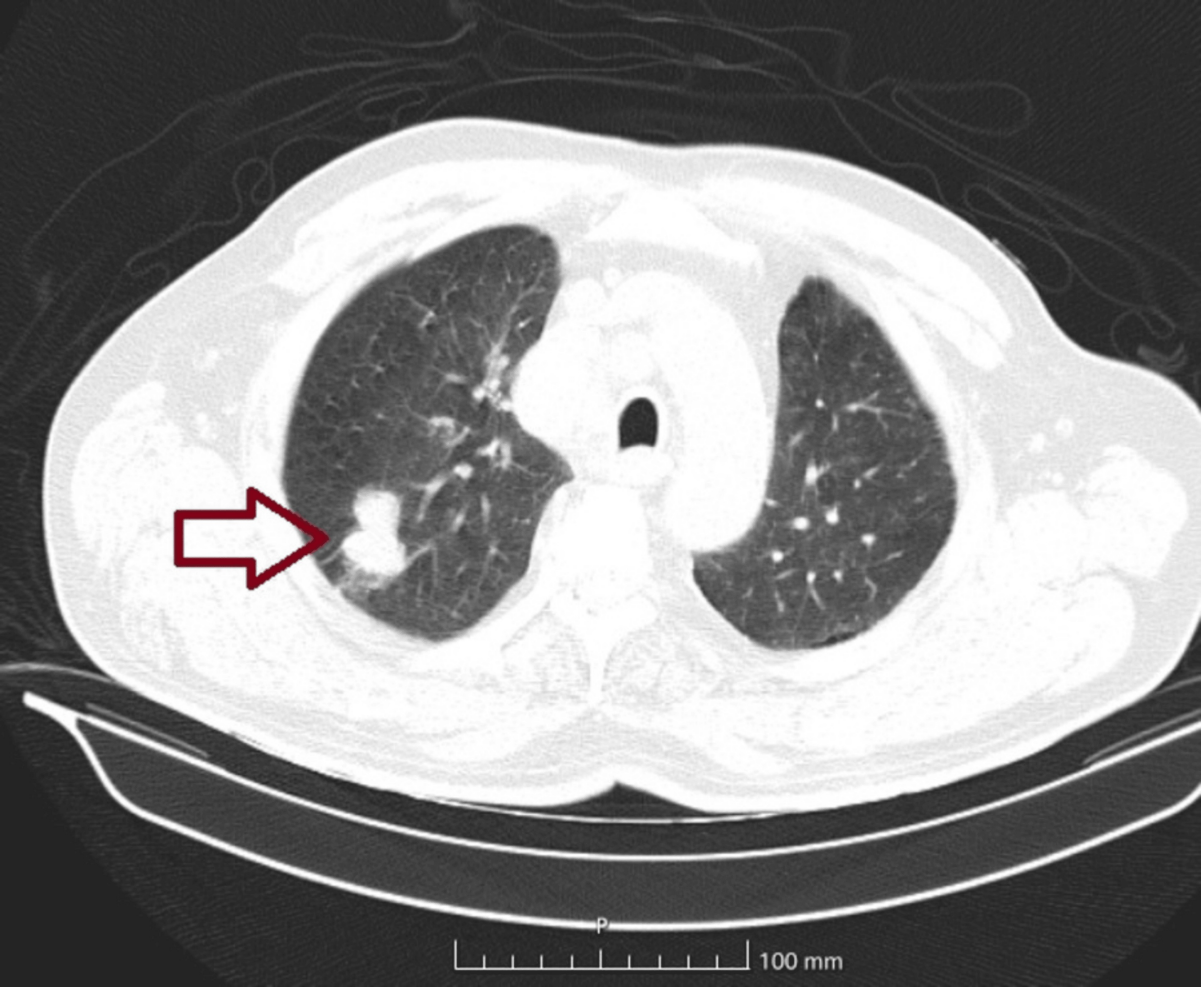
CT scan with a pulmonary mass in the right upper lobe.

Given the low probability of an infectious etiology, further investigations were conducted. A thoracoabdominopelvic CT scan revealed a 3.2 cm pulmonary mass in the right upper lobe, accompanied by a 1.4 cm satellite lesion, with no evidence of additional identifiable distant lesions (Figure 6).

**Figure 7:**
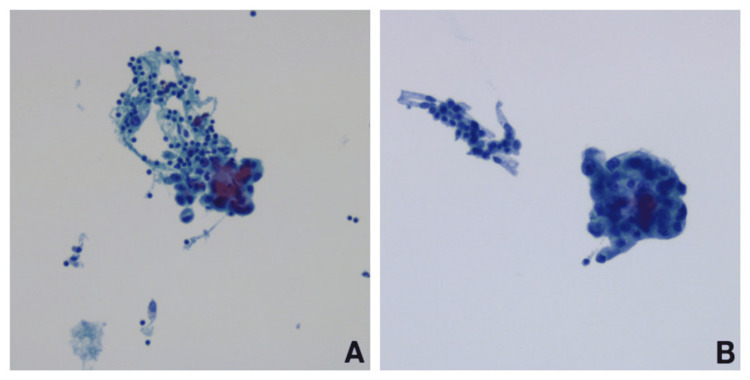
Cytological result of transcranial puncture (A) and transbronchial puncture (B) positive for neoplastic cells compatible non-small cell carcinoma. (A, transcranial) High cellularity, with a fibrin background, lymphocytes, occasional polymorphonuclear cells, bronchial cells, and numerous neoplastic cells, both isolated and in clusters, sometimes tridimensional, with an increased nucleus/cytoplasm ratio, dense cytoplasm, anisokaryosis, coarse chromatin, and prominent macronucleoli. (B, transbronchial): Moderate cellularity, composed of lymphocytes, occasional polymorphonuclear cells, numerous bronchial cells, and clusters of neoplastic cells, sometimes tridimensional, with dense cytoplasm, anisokaryosis, coarse chromatin, and prominent macronucleoli.

Bronchoscopy with transcranial and transbronchial biopsies revealed cytology positive for neoplastic cells consistent with non-small cell lung carcinoma. Immunocytochemical characterization was not possible due to insufficient material for cytoblock preparation (Figures 7A, 7B).

Following clinical stabilization, at the time of discharge, the patient had an Eastern Cooperative Oncology Group (ECOG) Performance Status of 3. Therapeutic anticoagulation was initiated, and the patient was referred for multidisciplinary care, including Oncology Pulmonology and Cardiology consultations, to evaluate the condition and consider endobronchial ultrasound (EBUS) for further neoplasm characterization, depending on functional status progression. One week after clinical discharge, the patient's neurological status worsened due to a new infarction in the right anterior cerebral artery territory, followed by nosocomial pneumonia with severe hypoxemia, ultimately culminating in death.

## Discussion

NBTE is a rare and underdiagnosed condition characterized by sterile vegetations composed of fibrin, platelets, and immune complexes. These vegetations predominantly involve the mitral valve, followed by the aortic valve, and have a significant propensity for systemic embolization, with ischemic stroke being a common clinical consequence [2,4]. Although NBTE is frequently identified postmortem, with an incidence ranging from 0.9% to 1.6% in the autopsy series, it remains a critical consideration in patients presenting with embolic phenomena [4,5]. Approximately 80% of cases are associated with advanced malignancies, while systemic lupus erythematosus accounts for most of the remaining cases [5].

Although cancer-associated NBTE is a rare condition, the most prevalent malignancies linked to it are those of the lung, pancreas and gastrointestinal tract [6]. While the precise pathophysiological mechanisms underlying NBTE remain incompletely understood, the prevailing hypothesis involves endothelial injury within a hypercoagulable state. Autopsy studies have not demonstrated metastasis in valvular vegetations but rather suggest that the hypercoagulable state associated with cancer plays a central role in NBTE development. The etiology of this hypercoagulability is multifactorial. The interaction between macrophages and tumor cells triggers the release of proinflammatory cytokines such as tumor necrosis factor (TNF), interleukin-1 (IL-1), and interleukin-6 (IL-6), which contribute to endothelial damage and cell detachment. This interaction between tumor cells (and tumor-derived substances such as cysteine proteases and tissue factor) and macrophages also triggers platelet activation, along with the activation of factor XII and factor X, leading to thrombin generation and thrombosis. [7]

The resulting embolization often leads to ischemic events, including stroke, as documented in cases of non-small cell lung cancer [8]. Ischemic stroke as the initial presentation of malignancy is rare, occurring in approximately 0.4% of cases [9].

Diagnosing NBTE can be challenging due to its overlap with infective endocarditis. The modified Duke criteria are crucial in excluding infective endocarditis by combining microbiological, clinical, and echocardiographic evidence. In this case, blood cultures were consistently negative, and the patient exhibited no signs of systemic infection, such as fever, leukocytosis, or elevated inflammatory markers like C-reactive protein. Transesophageal echocardiography revealed mobile, sterile vegetations on the mitral valve without evidence of abscess formation, structural destruction, or significant regurgitation, thereby supporting the diagnosis of NBTE [10].

A diagnostic triad described by McKay and Wahle, consisting of an underlying disease associated with NBTE, the presence of a heart murmur, and evidence of multiple systemic emboli, may further aid in identifying this condition. Importantly, a high index of clinical suspicion is essential. NBTE should be considered in patients presenting with acute stroke or coronary ischemia in the context of malignancy, systemic lupus erythematosus, or antiphospholipid syndrome. Furthermore, cases involving acute stroke with multiple, widely distributed embolic lesions of unknown etiology or presumed IE unresponsive to antibiotic therapy should prompt consideration of NBTE. Imaging findings may also provide diagnostic clues, as NBTE-related strokes typically present as multiple disseminated infarcts of varying sizes, contrasting with the more variable patterns associated with IE [5].

Treatment strategies for NBTE include systemic anticoagulation to reduce the risk of recurrent embolization and targeted management of the underlying malignancy. Surgical intervention may be necessary in select cases, although cancer-related NBTE often signifies advanced disease and carries a poor prognosis [11,12].

## Conclusions

In this case, NBTE presented as an unexpected initial manifestation of malignancy in a patient with ischemic stroke. The diagnosis was established through a detailed echocardiographic evaluation and the systematic exclusion of infectious causes. This highlights the importance of recognizing malignancy as a potential underlying cause in patients with embolic events, particularly in the presence of multiple embolic lesions or clinical features inconsistent with infective endocarditis. Early diagnosis, initiation of systemic anticoagulation, and appropriate oncologic therapy are critical to optimizing patient outcomes, even in the context of a poor overall prognosis associated with this condition.
